# Interactions of Peptide Amphiphiles With Viruses and Cells Are Enabled by Amorphous Nanostructures

**DOI:** 10.1002/psc.70051

**Published:** 2025-08-15

**Authors:** Julia La Roche, Lena Rauch‐Wirth, Laura Zimmerman, Fabian Zech, Jan Münch, Clarissa Read, Kübra Kaygisiz

**Affiliations:** ^1^ Central Facility for Electron Microscopy Ulm University Ulm Germany; ^2^ Institute of Molecular Virology Ulm University Medical Center Ulm Germany; ^3^ Department Synthesis of Macromolecules Max Planck Institute for Polymer Research Mainz Germany

**Keywords:** electron microscopy, retroviral infectivity, self‐assembly, transduction enhancer

## Abstract

Peptide amphiphiles can form fibrillar and amorphous structures. While fibrillar assemblies have previously been shown to enhance viral infectivity or retroviral transduction for gene delivery, we now elucidate the mechanism behind amorphous peptide amphiphiles that promote virus–cell interactions. Using electron microscopy, we reveal that amorphous fragments of polyunsaturated peptide amphiphiles allow for more VLPs to bind directly to the plasma membrane, explaining previously observed efficient viral entry and superior biodegradation compared to state‐of‐the‐art adjuvants. We believe our work highlights the potential of unsaturated fatty acid peptide hybrid materials for clinical applications.

## Graphical abstract

Amorphous nanostructures from polyunsaturated peptide amphiphiles such as eicosapentaenoyl‐VVVAAAKKK‐NH_2_ promote gene delivery by viral vectors.

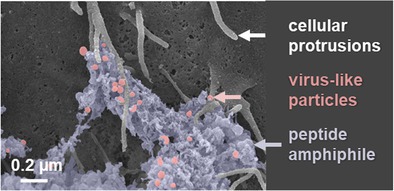

## 1 Introduction

Retroviral vectors are widely used in gene transfer and therapy applications [1]. However, a major challenge in the use of these viruses is their limited gene transduction efficiency, primarily due to inefficient virus attachment to the cell [2]. Transduction enhancers have been developed to improve virus attachment [3]. Among these, peptide‐based enhancers, especially self‐assembling positively charged peptides, have emerged as promising candidates [4, 5]. These materials function mainly by reducing electrostatic repulsion between the negatively charged viral and cell membrane [6] and consequently stimulating uptake by engaging with cellular protrusions [7, 8]. Recent studies have focused on optimizing the molecular structure of self‐assembling peptides to enhance their performance and safety in the context of retroviral gene transduction [9, 10]. For example, the self‐assembling D4 peptide, derived from in silico screening and structure–activity relationship analysis, shows improved transduction efficiency while forming biodegradable nanofibrillar aggregates that minimize immunogenicity and cytotoxicity [11].

In a previous study [9], we demonstrated that certain peptide amphiphiles (PAs), such as palmitoyl‐VVVAAAKKK‐NH_2_ (pal‐PA, termed PA1 in the previous study) and eicosapentaenoyl‐VVVAAAKKK‐NH_2_ (eic‐PA, termed Pat10 in the previous study), can enhance infection by retroviruses, such as the human immunodeficiency virus‐1 (HIV‐1), to levels comparable with commercially available transduction enhancers, but with improved degradability, making them promising adjuvants for clinical use.

PAs are a class of molecules that consist of a peptide part linked to an aliphatic group and are known for their ability to self‐assemble into various nanostructures [12, 13]. However, unlike existing transduction enhancers that rely on nanofibril formation such as pal‐PA to facilitate virus‐cell interactions [4, 10, 14], we previously found that eic‐PA can efficiently deliver viruses without the formation of fibrils and exhibits the highest cellular degradation efficiency observed to date (99%) [9]. While eic‐PA demonstrates remarkable performance, the underlying mechanism by which it interacts with virus particles and cells remains elusive.

In this communication, we present the first electron microscopic evidence of peptide amphiphile‐mediated virus‐cell interactions that do not rely on fibril formation but instead involve amorphous structures. By comparing the potent transduction enhancers pal‐PA and eic‐PA concerning their interaction with virus‐like particles (VLPs) and cells, we reveal that eic‐PA's mechanism of action involves binding to both the VLP membrane and the plasma membrane.

## 2 Morphologic Characterization Reveals Amorphous Nanostructure for Unsaturated Fatty Acid Peptide Amphiphile

**FIGURE 1:**
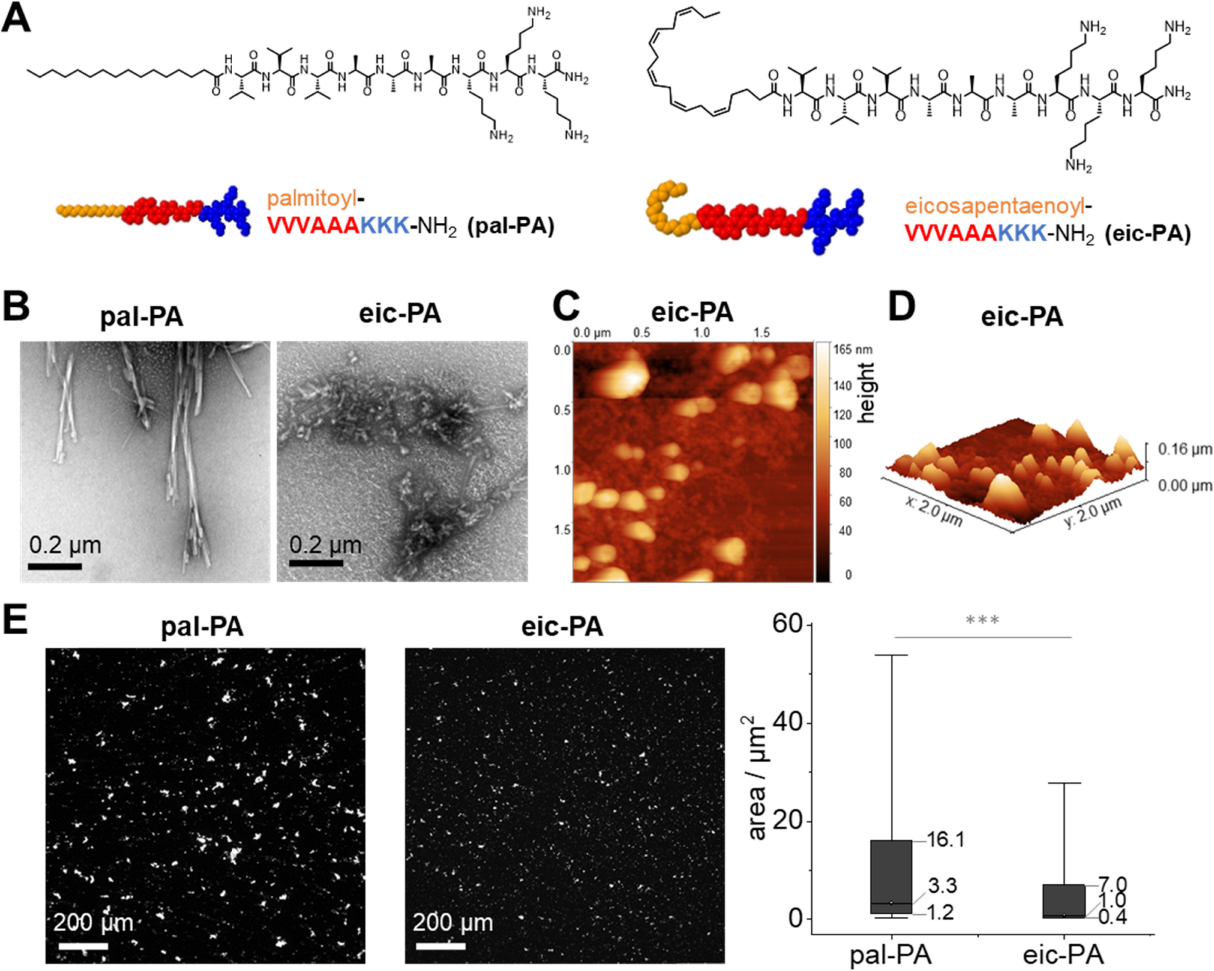
Morphologic characterization reveals spherical structure for the unsaturated fatty acid peptide eic‐PA. (A) Schematic illustration showing the sequence of the peptide amphiphiles palmitoyl‐VVVAAAKKK‐NH_2_ (pal‐PA, C_58_H_111_N_13_O_10_) and eicosapentaenoyl‐VVVAAAKKK‐NH_2_ (eic‐PA, C_62_H_109_N_13_O_10_), which were previously described [9]. (B) TEM images of peptide amphiphiles pal‐PA and eic‐PA, scale bar 0.2 μm. (C) AFM micrograph of eic‐PA, image width is 2 μm, color code represents z‐height. (D) AFM topology view of eic‐PA. (E) Quantification of micrometer structure of pal‐PA and eic‐PA via fluorescence microscopy, λ_em_ = 527 nm, λ_ex_ = 480 nm. The peptide structures were incubated at 0.1 mg/mL in PBS and stained with 50 μM Thioflavin‐T. Box plot summarizes micrometer‐sized structures observed in an area of 1300 μm × 1300 μm. While 23,709 aggregates have been counted for eic‐PA, pal‐PA contains at the same concentration approx. 5 times less aggregates (5063) in the same area. The box represents the 25th–75th percentile and the whiskers show the 10th–90th percentile, while the dots indicate the median. Statistical significance was assessed using the Mann–Whitney *U* test; *** indicates *p* < 0.0005. AFM: atomic force microscopy; TEM: transmission electron microscopy. Methods see Supporting Information.

The eicosapentaenoyl moiety of eic‐PA is a polyunsaturated fatty acid amide with a 20‐carbon chain and five cis double bonds. These double bonds introduce a kinked conformation (Figure 1A) that prevents dense packing and results in increased molecular flexibility and lower melting points compared to saturated fatty acids. We hypothesized that attaching an unsaturated fatty acid like eicosapentaenoic acid into a positively charged, β‐sheet prone peptide, such as VVVAAAKKK‐NH_2_, would increase structural disorder and disrupt self‐assembly into ordered fibrils [15]. Indeed, our results show that eic‐PA self‐assembles into amorphous structures of heterogeneous size, ranging from nanometers to micrometers, as observed through transmission electron microscopy (TEM, Figure 1B) and atomic force microscopy (AFM, Figure 1C–D, methods see Supporting Information). Despite the amorphous morphology at the nanoscale, the assemblies retain characteristic β‐sheet features at the molecular level that are observed via infrared absorption peak at 1630 cm^−1^ and binding of β‐sheet–specific dyes such as Thioflavin‐T and Proteostat, as previously demonstrated [9]. The micrometer‐sized aggregates formed by eic‐PA have a median area of 1.0 μm^2^, which is significantly smaller than those formed by pal‐PA, with a median area of 3.3 μm^2^ (Figure 1E). This difference in size corresponds with the number of aggregates observed as eic‐PA forms approx. 5 times more aggregates (23,709) than pal‐PA (5063). Overall, the higher number and smaller size of β‐sheet rich eic‐PA aggregates are expected to enhance VLP delivery to cells by providing a larger surface area and binding sites for interaction with both VLPs and cells.

## 3 Interaction of Peptide Amphiphiles With Cells and VLPs

**FIGURE 2:**
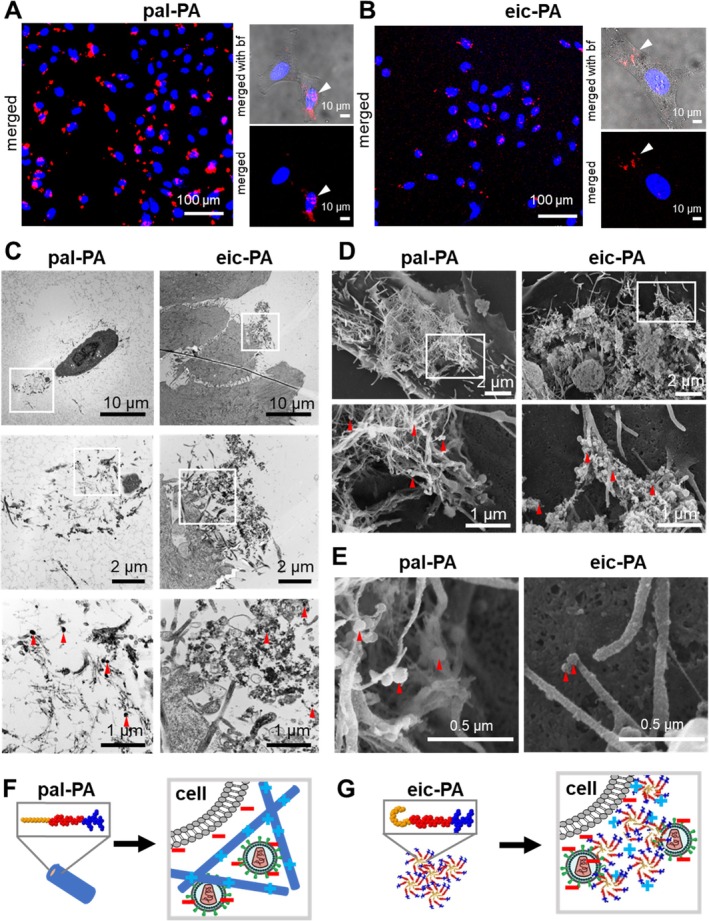
Interaction of PAs with cells and virus‐like particles. (A and B) Confocal fluorescence microscopy image of (A) pal‐PA and (B) eic‐PA shows the colocalization of PA clusters with cells. HeLa cells were incubated with PAs for 30 min at 37°C. PAs are stained with proteostat (red), and cell nuclei are stained with Hoechst 33342 (blue), scale bar 100 μm. Right panels show higher magnifications of the PA‐cell interaction (white arrowheads) with and without brightfield merged channels, scale bar 10 μm. (C) TEM images of pal‐PA and eic‐PA clusters interacting with HeLa cells and VLPs (red arrowheads) after 1 h of incubation. White boxes highlight magnified areas below. (D) SEM images of pal‐PA and eic‐PA interacting with HeLa cells and VLPs (red arrowheads). (E) SEM image highlighting VLPs (red arrowheads) directly bound to small eic‐PA fragments and to the cell body compared to VLPs bound to fibers from pal‐PA. (F) Schematic illustration showing fibrillar pal‐PA clusters and (G) amorphous eic‐PA clusters interacting with VLPs and cells. Scheme not true to scale. bf: brightfield; PA: peptide amphiphile; SEM: scanning electron microscopy; TEM: transmission electron microscopy; VLPs: virus‐like particles. Methods see Supporting Information.

To understand how eic‐PA's amorphous structure enhances HIV‐1 binding to cells, we compared pal‐PA (fibrillar structures) with eic‐PA (amorphous structures) concerning their interaction with VLPs developed from murine leukemia virus (MLV) and cells using confocal light microscopy and electron microscopy. We used these VLPs as a safe alternative for HIV‐1 as they show comparable morphology and uptake but are non‐infectious [16]. Initially, we hypothesized that both PAs interact with cells and VLPs in a manner similar to previously studied amyloid‐type fibrils, by bringing VLPs closer to the cell membrane, particularly at cellular protrusions [7]. Confocal microscopy revealed that micrometer‐sized PA aggregates colocalize with cells within 30 min of incubation (Figure 2A,B and Video S1). Notably, the aggregates formed by eic‐PA were more evenly distributed in the culture dish and smaller in size compared to the larger pal‐PA.

To gain further insights, we performed TEM (Figure 2C) and scanning electron microscopy (SEM, Figure 2D) and confirmed the fibrillar morphology for pal‐PA and amorphous morphology for eic‐PA and attachment of VLPs to both (red arrowheads in Figure 2C–E, methods see Supporting Information). Furthermore, imaging revealed the interaction of PA‐VLP‐aggregates with cellular protrusions. SEM further confirmed that pal‐PA forms larger aggregates than eic‐PA. VLPs seem to bind pal‐PA within its large mesh‐like fibrillar aggregates, whereas they rather bind the surface of the amorphous eic‐PA aggregates. Both PA types were found in close proximity to filopodia and the plasma membrane in general. Moreover, we expected that amorphous eic‐PA oligomers are also more likely to directly attach to the surface of VLPs and cover their surface compared to fibrillar pal‐PA structures. We observed for eic‐PA that the surface of VLPs (Figure 2E, red arrowheads) and of filopodia (Figure 2E) looked slightly less smooth compared to pal‐PA samples, suggesting that small eic‐PA fragments are covering the VLP and cell surface. This characteristic of eic‐PA likely makes it a more efficient transduction enhancer, as it bridges the interaction between VLPs and the plasma membrane. From this, we conclude that VLPs associated with amorphous eic‐PA structures, either the micrometer large aggregates or the small fragments, can bind directly to the plasma membrane (Figure 2G). Such direct interaction between VLPs and the cell membrane is rarely observed with the fibrillar pal‐PA structures, where VLPs seem to be often trapped within the mesh‐like fibril aggregate (Figure 2F). We hypothesize that the direct binding of VLPs to the plasma membrane by eic‐PA enhances the likelihood of VLP uptake and gene delivery. Overall, we believe that this makes amorphous peptide nanostructures, as shown here with eic‐PA, promising candidates for therapeutic applications.

More broadly, our findings contribute to an understanding of how nanostructure morphology influences biological function. While the design rules for fibrillar peptide assemblies are well established and mainly driven by directional hydrogen bonding, amorphous peptide assemblies remain comparatively underexplored [17]. In contrast to the structural rigidity of fibrils, loosely aggregated structures can offer higher molecular accessibility, which can enable more dynamic interactions and improved uptake with cellular membranes [18]. In this context, molecular design strategies for disordered nanomaterials, such as balancing hydrophobic and electrostatic interactions [19], using C3‐branched scaffolds [20], or leveraging membrane‐induced peptide aggregation [21], have shown promise in enhancing cellular uptake. Here, we have demonstrated that combining an unsaturated lipid with a β‐sheet–prone peptide like eic‐PA leads to the formation of amorphous nanostructures with enhanced membrane binding and transduction efficiency and overcomes key limitations of rigid fibrillar systems, such as low biodegradability. Future work could explore how different lipid–peptide combinations influence nanoscale structure and improve delivery outcomes across various cell types and cargos.

## Conflicts of Interest

L.R‐W., J.M., and K.K. are inventors of granted and filed patents for the usage of peptide amphiphiles as degradable enhancers of retroviral gene transfer.

## Supporting information


**Data S1:** Supporting information.

**Video S1:** Supporting information.

## Data Availability

The data that support the findings of this study are available from the corresponding author upon reasonable request.
