# Supplementary material for: Interactions of Peptide Amphiphiles With Viruses and Cells Are Enabled by Amorphous Nanostructures
Source: J Pept Sci. 2025 Aug 15;31(9):e70051. doi: 10.1002/psc.70051 (PMC12355531; doi:10.1002/psc.70051)
Supplement: Supplementary file 1 — Data S1: Supporting information. [file PSC-31-e70051-s002.docx]

**SUPPORTING INFORMATION**

**Interactions of Peptide Amphiphiles with Viruses and Cells are Enabled by Amorphous Nanostructures**

Julia La Roche^1^, Lena Rauch-Wirth^2^, Laura Zimmerman^2^, Fabian Zech^2^, Jan Münch^2^, Clarissa Read^1^, Kübra Kaygisiz^3‡^

1 Central Facility for Electron Microscopy, Ulm University, Ulm, 89081, Germany

2 Institute of Molecular Virology, Ulm University Medical Center, Meyerhofstraße 1, 89081 Ulm, Germany

3 Department Synthesis of Macromolecules, Max Planck Institute for Polymer Research, Ackermannweg 10, 55128 Mainz, Germany

‡ Corresponding author, present address: Department of Chemistry, Massachusetts Institute of Technology, Cambridge, Massachusetts 02139, United States

**Experimental Section**

**Materials**

Palmitoyl–VVVAAAKKK-NH_2_ (pal-PA) was synthesized according to fluorenylmethyloxycarbonyl (Fmoc) solid phase peptide synthesis (SPPS) strategy by Merrifield using Rink Amide AM Resin LL and has been reported in a previous study.^[1]^ Eicosapentaenoyl–VVVAAAKKK-NH_2_ (eic-PA) was purchased from Bachem AG (> 87% purity) and has been reported in a previous study.^[1]^

Dulbecco's Phosphate Buffered Saline without Magnesium and Calcium (DPBS) was purchased from Sigma-Aldrich. Proteostat was purchased from Enzo Life Sciences. HeLa cells were cultured in Dulbecco´s Modified Eagle Medium (DMEM, Gibco, Germany) with 10% (v/v) fetal calf serum (FCS), L-glutamine (2 mM), penicillin (100 units/mL) and streptomycin (100 µg/mL). VLPs (Murine leukemia virus (MLV) with the gag protein fused to yellow fluorescent protein (YFP) (MLV gag-YFP) have been prepared according to a previous protocol.^[2]^

**Sample preparation**

Stock solutions were prepared by dissolving peptide amphiphiles in DMSO (10 mg/mL) and were stored at -20°C. Self-assembly was initiated by diluting the stock solution to 1 mg/mL in DPBS and incubated for 1 day at room temperature. Other concentrations were obtained by further diluting the preformed fibrils in DPBS.

**Fluorescence microscopy**

1 µL of the prepared peptide nanostructures in PBS (1 mg/mL) were added to 9 µL, 50 µM ThT in PBS to yield a 0.1 mg/mL peptide solution. 10 uL of this solution was placed on a microscopy glass and fluorescence microscopy was performed using a Leica DMi8 microscope with 10x air objective and a Leica MC170 HD camera with a filter setting λ_em_ = 527/30 nm, λ_ex_ = 480/40 nm. Size and number of Thioflavin-T-stained aggregates were determined using ImageJ 1.53f51 “Analyze Particles” function. The microscope images cover an area of 1,300 µm × 1,300 µm. The area detection limit for a single aggregate was 0.41 µm^2^.

**Atomic force microscopy**

Atomic force microscopy (AFM) was performed in dry state using a Bruker Dimension FastScan Bio atomic force microscope. AFM probes with a nominal force constant of 26 N/m and a resonance frequency of 300 kHz (OTESPA-R3, Bruker) were used and operated in tapping mode. Samples were prepared by lyophilizing pre-formed peptide amphiphiles onto a mica substrate. The obtained measurements were processed using Gwyddion.

**Confocal microscopy**

HeLa cells were seeded in an 8-well IBIDI slide at a density of 40,000 cells per well one day before the assay. Preformed PA (1 mg/mL) were mixed with 4 µL of Proteostat (1 µL stock in 999 µL PBS) and incubated for 10 minutes. The solution was then further diluted with DMEM to achieve a final peptide concentration of 20 µg/mL. The HeLa cell nuclei were stained with Hoechst 33342 (NucBlue, Thermo Fisher Scientific). The peptide solution was added to the cells and incubated for 30 minutes at 37°C, followed by three washes with PBS. Peptide aggregate interaction with the cells was monitored after the 30-minute incubation using a Stellaris 8 confocal laser scanning microscope (Leica) equipped with a 20x air objective and laser excitation wavelength of 405 nm (Hoechst) and 561 nm (Proteostat).

**Transmission electron microscopy (TEM)**

For visualization of PAs only, 10 µl of pre-formed peptide aggregates (2 mg/ml) were incubated on a glow-discharged Formvar film on a 300-mesh copper grid (Plano GmbH) for 10 min. Samples were washed on three drops of aqua bidest and negatively stained with a drop of 2 % (w/v) uranyl acetate (Merck) for 5 s. Before air drying, most of the uranyl acetate solution was removed using filter paper (VWR). PAs were imaged at 120 kV acceleration voltage using a JEM-1400 transmission electron microscope (JEOL).

To visualize the interaction of PAs with VLPs and HeLa cells, 50,000 HeLa cells were seeded on glow-discharged carbon-coated sapphire disks (3 mm diameter, 50 μm thickness, Wohlwend GmbH) in a 12 well plate. The next day PAs (2 µg/ml) mixed with VLPs (250 µg/ml) for 10 min were added to cells for 1 h at 37°C (1:200 diluted on cells). Samples were, as described previously,^[3,4]^ high-pressure frozen (HPF Compact 01, Wohlwend GmbH), freeze substituted (EM AFS2, Leica) and embedded in epoxy resin. The embedded samples were ultrathin sectioned (thickness of about 70 nm) and imaged with a JEM-1400 transmission electron microscope (JEOL) at 120 kV.

**Scanning electron microscopy (SEM)**

Cell culture and incubation of cells with PAs and VLPs was performed identical to sample preparation for TEM. Then, cells were fixed overnight at 4 °C using 2.5 % glutaraldehyde, 1 % saccharose (VWR) in 0.1 M phosphate buffer, pH 7.3. Samples were stained and post-fixed with 2 % osmium tetroxide (ChemPur Feinchemikalien und Forschungsbedarf GmbH) at room temperature for 20 min. After three times washing with PBS, samples were dehydrated in increasing concentrations of propanol. Critical point dried (CPD 030, BAL-TEC AG) samples were coated with about 5 nm platinum (BAF 300, BAL-TEC AG) and imaged at 5 kV using a Hitachi S-5200 field emission scanning electron microscope (Hitachi).^[5]^

**References**

[1] K. Kaygisiz, L. Rauch‐Wirth, A. Iscen, J. Hartenfels, K. Kremer, J. Münch, C. V. Synatschke, T. Weil, *Adv. Healthcare Mater.* **2024**, *13*, 2301364.

[2] L. Rauch-Wirth, D. Schütz, R. Groß, S. Rode, B. Glocker, J. A. Müller, P. Walther, C. Read, J. Münch, *Biomaterials* **2025**, *317*, 123044.

[3] P. Walther, A. Ziegler, *J. Microsc.* **2002**, *208*, 3.

[4] M. Schauflinger, C. Villinger, T. Mertens, P. Walther, J. von Einem, *Cell. Microbiol.* **2013**, *15*, 305.

[5] B. Beitzinger, F. Gerbl, T. Vomhof, R. Schmid, R. Noschka, A. Rodriguez, S. Wiese, G. Weidinger, L. Ständker, P. Walther, J. Michaelis, M. Lindén, S. Stenger, *Adv. Healthcare Mater.* **2021**, *10*.
